# Biological Activity of Some Cobalt(II) and Molybdenum(VI) Complexes: *in vitro* Cytotoxicity

**DOI:** 10.1155/S1565363304000123

**Published:** 2004

**Authors:** Nikos Katsaros, Maria Katsarou, Sofija P. Sovilj, Ksenija Babić-Samardžija, Dragana M. Mitić

**Affiliations:** 1 N.C.S.R. “Demokritos ” Institute of Physical Chemistry Agia Paraskevi Attikis 15 310 Greece; 2 Faculty of Chemistry University of Belgrade P. O. Box 158 Belgrade 11001 Serbia and Montenegro; 3 Faculty of Stomatology Dr. Subotića 8 Belgrade 11000 Serbia and Montenegro

## Abstract

Cytotoxicity and cell growth inhibition studies were performed for five distinct cobalt(ll) [Co_2_(acac)tpmc](ClO_4_)_3_, [Co_2_(dibzac)tpmc](ClO_4_)_3_, [Co_2_(hfac)tpmc](CIO_4_)_2_, [Co_2_(tmhd)tpmc](CIO_4_)_3_ and [Co_2_(ox)tpmc](CIO_4_)_2_.3H_2_0 and five molybdenum(Vl) complexes, [MoO_2_(pipdtc)_2_], [MoO_2_(morphdtc)], [MoO_2_(timdtc)_2_], [MoO_2_(pzdtc)_2_] and [MoO_2_(N-Mepzdtc)_2_]. The former were tested in two leukemia cell lines: chronic myelogenic leukemia (K562) and human promyelocytic cell line (U937). They showed to have relatively high toxicity in K562 cells and a relatively low cytotoxicity in U937 cells, as assessed by both MTT and Trypan Blue assays. The five molybdenum complexes were tested in human promyelotic U937 cell line and they showed to have high toxicity.


### Biological Activity of Some Cobalt(II) and Molybdenum(VI) Complexes: *in vitro* Cytotoxicity

Nikos Katsaros[a*], Maria Katsarou[a], Sofija P. Sovilj[b], Ksenija Babić-Samardžija[b] and Dragana M. Mitić[c]

[a] *N.C.S.R. "Demokritos ", Institute of Physical Chemistry, 15 310 Agia Paraskevi, Attikis, Greece, Phone:+302106503645, Fax: +30210651176, E-mail: katsaros@chem.demokritos.gr*
[b] *Faculty of Chemistry, University of Belgrade, P. O. Box 158, 11001 Belgrade, and*
[c] *Faculty of Stomatology, Dr. Subotića 8, 11000 Belgrade, Serbia&Montenegro*

### ABSTRACT

Cytotoxicity and cell growth inhibition studies were performed for five distinct cobalt(II) $[Co_2(acac)tpmc](ClO_4)_3$, $[Co_2(dibzac)tpmc](ClO_4)_3$, $[Co_2(hfac)tpmc](ClO_4)_3$, $[Co_2(tmhd)tpmc](ClO_4)_3$ and $[Co_2(ox)tpmc](ClO_4)_2 \cdot 3H_2O$ and five molybdenum(VI) complexes, $[MoO_2(pipdtc)_2]$, $[MoO_2(morphdtc)_2]$, $[MoO_2(timdtc)_2]$, $[MoO_2(pzdtc)_2]$ and $[MoO_2(N\text{-}Mepzdtc)_2]$. The former were tested in two leukemia cell lines: chronic myelogenic leukemia (K562) and human promyelocytic cell line (U937). They showed to have relatively high toxicity in K562 cells and a relatively low cytotoxicity in U937 cells, as assessed by both MTT and Trypan Blue assays. The five molybdenum complexes were tested in human promyelotic U937 cell line and they showed to have high toxicity.

### 1. INTRODUCTION

In order to understand the mechanisms of action of chemicals on cells and tissues it is important to perform cytotoxicity tests, since the cytotoxicity of a compound is thought to play an important role in a number of pathological processes, including carcinogenesis and inflammation /1/. The use of cell culture systems has become common in the toxicological assessment of chemicals and chemical mixtures. Various in vitro cytotoxicity assays have been evaluated by large research groups all over the world in order to prove their importance in toxicology /2/. What is certain is that in vitro testing minimizes the need for animal use in toxicity assessments, while it is useful in assessing the toxicity of new products in the early stages of development.

Two *in vitro* citotoxicity assays were selected, five dinuclear cobalt(II) complexes (**I-V**) with macrocyclic ligand *tpmc* (N,N',N'',N'''-tetrakis(2-pyridylmethyl)-1,4,8,11-tetraazacyclotetradecane) and one of the additional bidentate i.e., 2,4-pentanedionato (*acac*), 1,3-diphenyl-1,3-propanedionato (*dibzac*), 1,1,1,5,5,5-hexafluoro-2,4-pentanedionato (*hfac*), 2,2,6,6-tetramethyl-3,5-heptanedionato (*tmhd*) or oxalate (*ox*) ions, and five molybdenum(VI)-dioxo complexes (**A-E**) with *pipdtc*, *morphdtc*, *timdtc*, *pzdtc* and *N-mepzdtc* ligands that refer to piperidine-, 4-morpholine-, 4-thiomorpholine-, piperazine- and N-methylpiperazine-dithiocarbamates, respectively, for use in the study. The viability of cells was determined by the Trypan Blue dye exclusion method and cytotoxicity was assessed by the MTT assay.

### 2. EXPERIMENTAL

### 2.1. Materials

The reagents RPMI 1640 and Fetal Bovine Serum (FBS), the antibiotics penicillin, streptomycin and the buffers Phosphate Buffered Saline (PBS) and HEPES were purchased from BIOCHROM. L-glutamine was purchased from AppliChem. Trypan Blue, 3-(4,5-dimethylthiazol-2-yl)-2,5-diphenyl-tetrazoliumbromide (MTT), sodium-dodecyl sulfate (SDS), $NaHCO_3$, formamide, dimethylsulfoxide (DMSO) and methanol were purchased from Sigma-Aldrich.

The syntheses of the cobalt(II) complexes (**I-IV**) were described elsewhere /3/ as well as the synthesis, structure and properties of the complex $[Co_2(ox)tpmc](ClO_4)_2{\cdot}3H_2O$ (**V**) /4/ and the molybdenum(VI) complexes (**A-E**) in literature /5/.

### 2.2. Cell cultures

Two human cell lines routinely maintained in the Biology Department of NCSR "Demokritos" were used in these studies. Chronic myelogenic leukemia (K562) and human promyelocytic (U937) cell lines were maintained in RPMI 1640 medium containing 10% (*v/v*) FBS, 2 mM L-glutamine, 0.85 g/l $NaHCO_3$, 25 mM HEPES, 200 U/ml penicillin and 100 μg/ml streptomycin at 37 °C in a 5% $CO_2$ atmosphere. The pH of the medium was kept 7.3 by use of HCl. In all experiments, all the cells used were cultured to logarithmic phase growth over a period of 48 h starting from a culture density $4{\cdot}10^5$ cells/ml.

### 2.3. Cell viability and cytotoxicity

The viability of cells was determined by the Trypan Blue dye exclusion method and cytotoxicity was assessed by the MTT assay /6-9/. Briefly, logarithmically grown cells were plated in 25 $cm^2$ flasks and treated with two concentrations of each cobalt compound dissolved in DMSO and with two concentrations of each molybdenum complex dissolved in methanol.

Control cells were treated with DMSO alone and positive controls with various amounts of five cobalt complexes (**I-V**). Control cells were treated with methanol alone and positive controls with various amounts of five molybdenum complexes (**A-E**). Untreated cells were used as a negative control before each experiment. In more detail, before the addition of the compounds, the toxicity of the two solvents alone in the cell lines was tested. For the compounds (**I-V**) two volumes of DMSO were tested: One that gives 100 μg/ml concentration of each compound in solution and one that gives 10 μg/ml concentration. The same test was done for the toxicity of methanol. It was found that the toxicity of both the solvents was not significant (>80% alive cells ). As a result, the maximum volume of each compound in DMSO or methanol that could be added in the cell culture was 150 μl. Due to this limitation and taking into account that the compounds had different solubilities in the solvents used, the concentrations of the compounds that were tested differed.

Incubation was carried out at 37 °C for different time periods, starting from 24 h of incubation up to 72 hours. After drug incubation for various time periods at 37 °C, 50 μl of MTT (1 mg/ml, Sigma) was added to each well followed by 4 h incubation at temperature of 37 °C. The reaction results in the reduction of MTT by the mitochondrial dehydrogenases of viable cells to a purple formazan product. Cells were lysed, using DMF solution (55 ml $H_2O$ + 12.5 g SDS + 45 ml formamide, at pH 4.7) and left overnight. After centrifugation, 200 μl of each sample was placed in 96-well microtiter plates in duplicate and $OD_{550\ nm}$ was determined in an ELISA plate reader. The results were expressed as the percentage of alive cells as calculated from MTT reduction, assuming the absorbance of control cells as 100%. The 50% inhibitory concentration (concentration of drug required to inhibit cell growth by 50%, $IC_{50}$) was calculated for each complex tested from dose-response curves for incubation periods of 24, 48 and 72 h for U937 cells, and 24 and 48 h for K562 cells for cobalt compounds, and 24, 50 and 72 h for molybdenum compounds. In addition, cell viability in U937 and K562 cell lines was assessed by the method of Trypan Blue exclusion. Trypan Blue (0.2%, 2 μl) was added to aliquots of cell-containing media (18 μl) and the percentage of viable cells (-ve cells) was determined by counting on a hemocytometer the number of cells able to exclude the dye.

### 3. RESULTS AND DISCUSSION

### 3.1. Cobalt complexes

Each tumor cell line was treated with five different cobalt compounds, namely $[Co_2(acac)tpmc](ClO_4)_3$ (**I**), $[Co_2(dibzac)tpmc](ClO_4)_3$ (**II**), $[Co_2(hfac)tpmc](ClO_4)_3$ (**III**), $[Co_2(tmhd)tpmc](ClO_4)_3$ (**IV**) and $[Co_2(ox)tpmc](ClO_4)_2{\cdot}3H_2O$ (**V**) and viability was determined using the MTT method as well as the Tryphan Blue dye exclusion procedure. Figure 1 represents the cell density variation as a function of the incubation time of the cells with the five distinct cobalt(II) complexes tested, for different concentrations in U937 cell line, and in Table S1 (Supplementary Material) are the corresponding data. The dose-dependent cytotoxic effects on K562 leukemia cells of those compounds, after 24 and 48 hours of incubation, are displayed in Figure 2.

By analyzing these data, it is clear that all of the cobalt compounds (**I-V**) tested have relatively high toxicity when introduced in U937 cells after 72 h of immersion, besides the complex **V** (Fig. 1). In fact, data obtained from MTT reduction assay showed that complex **V** is the least toxic from all of the cobalt compounds examined, with higher values of alive cells after 24-72 h of incubation. Complexes **I-IV** have high toxicity in U937 cells for concentrations equal or higher than 10 μM in 72 h time. These complexes exhibited almost similar toxicity, which decreased when lower concentrations were used and increased when the compounds were left in the cells for longer time. Also, it is evident from the results (Fig. 1) that no definite trend was observed in the shift of cytotoxicity effect values but apparently the complex **II** is the most toxic for the U937 cells.

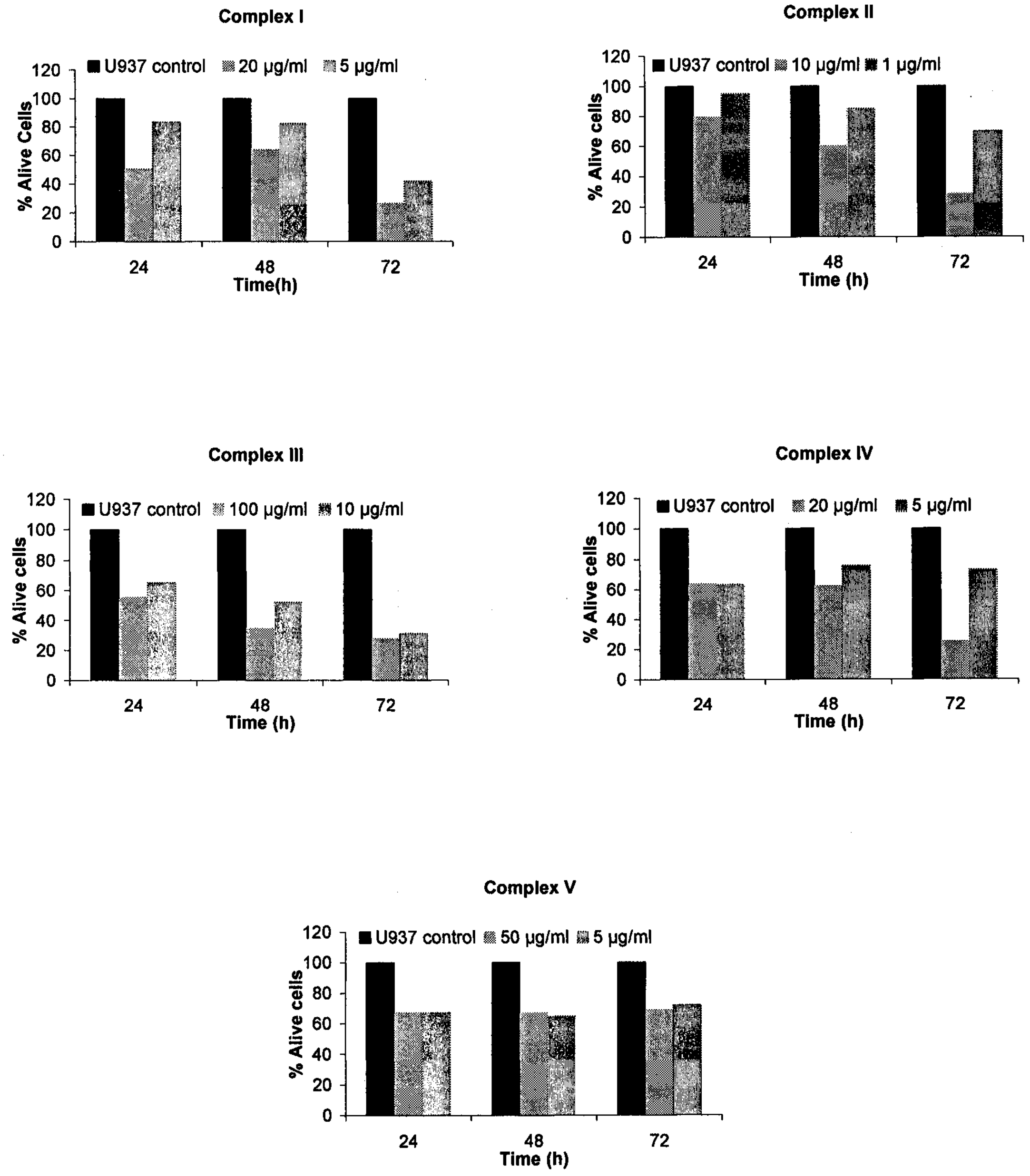

**Fig. 1:** Time- and dose-dependent cytotoxic effects of the complexes **I-V** on U937 cells. 10-12·$10^5$ cells/ml were incubated with the drugs for 24, 48 and 72 hours. Every 24 h aliquot of the cell suspensions were removed and cell viability was evaluated by the MTT colorimetric assay (as described in Section 2.). The data are expressed as a percentage of the control MTT reduction (100%).

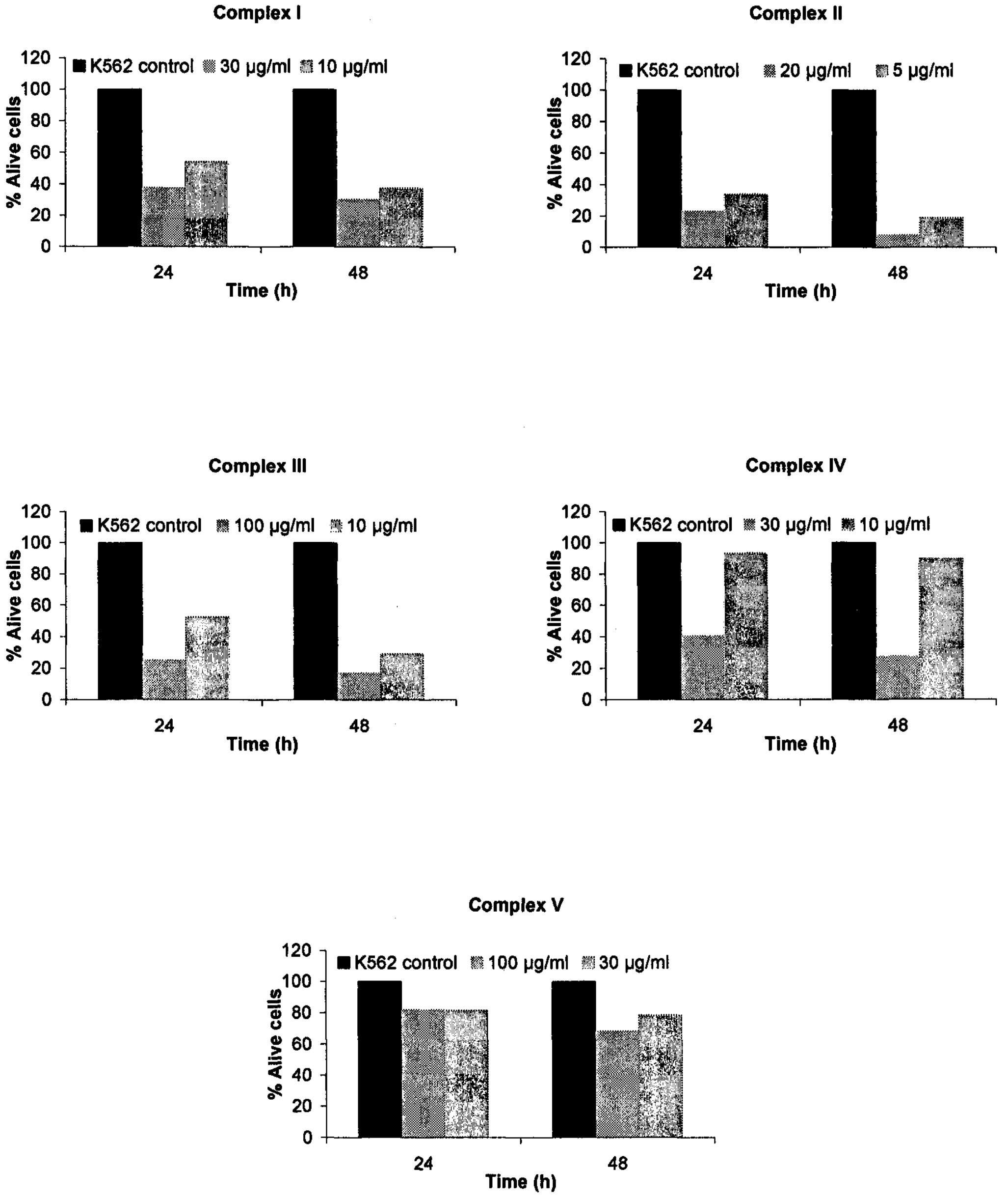

**Fig. 2:** Time- and dose-dependent cytotoxic effects of the complexes **I-V** on K562 cells. $10\text{-}12\cdot10^5$ cells/ml were incubated with the drugs for 24 and 48 hours. Every 24 h aliquot of the cell suspensions were removed and cell viability was evaluated by the MTT colorimetric assay (as described in Section 2.). The data are expressed as a percentage of the control MTT reduction (100%).

In the case of the compounds introduced in K562 cells line the effect of relatively high toxicity, when the cells were incubated for 24 and 48 h, in the presence of one of the cobalt complexes was found (Fig. 2). K562 cell line also showed low sensitivity to complex **V**. For concentrations of 10 μM or higher compounds **I, III** and **IV** might be considered to have significant cytotoxic and antiproliferative properties, while the complex **II** displays the highest cytotoxic activity for concentrations higher than 5 μM.

The results from the Table S1 (Supplementary Material) for the MTT assay show that $IC_{50}$ values for the cobalt complexes in U937 cells were approximately 20 μM (for **I**) < 100 μM (for **III**) < 20 μM (for **IV**) < 50 μM (for **V**) in 24 h and 10μM (for **II**) after 48 h of incubation time. The results presented in Table S2 (Supplementary Material) show that the $IC_{50}$ values for the cobalt complexes in K562 cells were about 5 μM (for **II**) < 30 μM (for **IV**) < 10 μM (for **III**) < 10 μM (for **I**) for the incubation time of 24 h, and lower then 30 and 100 μM (for **V**) after 24 and 48 h of immersion, respectively.

Tables 1 and 2 present the percentage of Trypan Blue (-ve) cells in U937 and K562 cell lines, respectively. The data shows that the number of alive (unstained) cells in 24 and 48 h for U937 and K562 cells for all the complexes in average higher then 80%, which means that none of the complexes exhibit significant necrotic effect on the cell lines tested. Increasing of incubation time and concentration of investigated compounds decreased the value of alive cells.

The results from both cell viability methods used in order to determine the toxicity of the cobalt(II) complexes indicate that the compounds exhibit lower toxicity in U937 cell line and more toxicity in K562 cells. Low toxicity in U937 cells is observed during the first 24 hours of immersion, and relatively high (after 48 h) and high toxicity when they were incubated for 72 h in the cells. This could be due to a lot of factors such as induction of apoptosis or differentiation. Low toxicity in K562 cells during 24 h and relatively high toxicity during the 48 h is noted for all of the complexes investigated. Also, the percentage of alive cells derived from Trypan Blue dye exclusion procedure is high in both cell lines which means that the toxic effect of the Co(II) complexes (**I-V**) on U937 and K562 leukemia cell lines is relatively low, especially after 24 h of incubation time.

### 3.2. Molybdenum complexes

Human promyelocytic cell line U937 was treated with two different concentrations of each molybdenum complexes, namely $[MoO_2(pipdtc)_2]$ (**A**), $[MoO_2(morphdtc)_2]$ (**B**), $[MoO_2(timdtc)_2]$ (**C**), $[MoO_2(pzdtc)_2]$ (**D**) and $[MoO_2(N\text{-}Mepzdtc)_2]$ (**E**), and viability was determined using the MTT method as well as the Tryphan Blue dye exclusion procedure. Figure 3 represents the cell density variation as a function of the incubation time of the cells with the five distinct molybdenum(VI)-dioxo complexes tested, for two different concentrations in U937 cell line.

Molybdenum complexes were dissolved and tested in methanol solutions, which is not a common solvent for compounds, which are introduced in cells. For this reason, control cells were treated with methanol alone in order to see whether methanol alone is toxic for the cells. The results from both the assays performed (MTT and Trypan Blue) showed that methanol does not have high toxicity on its own and that the number of necrotic cells is low.

**Table S1**

Cytotoxicity in U937 leukemia cell lines induced by exposure for 24, 48 and 72 h to different concentrations of the complexes I-V as assessed by MTT reduction

| Complexes I-V / Concentration | *% Alive cells*[b] | | |
|---|---|---|---|
| | **24h** | **48h** | **72h** |
| **I** | | | |
| 20 μg/ml | 50.4 ± 0.2 | 64.4 ± 0.1 | 26.9 ± 0.7 |
| 5 μg/ml | 83.8 ± 0.2 | 82.5 ± 0.3 | 41.8 ± 0.8 |
| **II** | | | |
| 10 μg/ml | 79.5 ± 0.1 | 60.3 ± 0.1 | 28.4 ± 0.2 |
| 1 μg/ml | 95.1 ± 0.1 | 85.1 ± 0.1 | 69.9 ± 0.1 |
| **III** | | | |
| 100 μg/ml | 55.5 ± 0.1 | 34.5 ± 0.5 | 27.9 ± 0.1 |
| 10 μg/ml | 65.4 ± 0.6 | 52.1 ± 0.8 | 31.3 ± 0.2 |
| **IV** | | | |
| 20 μg/ml | 63.9 ± 0.02 | 62.5 ± 0.004 | 25.5 ± 0.002 |
| 5 μg/ml | 63.3 ± 0.06 | 75.8 ± 0.03 | 73.1 ± 0.005 |
| **V** | | | |
| 50 μg/ml | 67.5 ± 0.005 | 66.9 ± 0.001 | 69.1 ± 0.003 |
| 5 μg/ml | 67.5 ± 0.004 | 64.8 ± 0.003 | 72.3 ± 0.006 |

[b] % alive cells were determined using the following formula: %alive cells = (mean absorption of sample 100) / mean absorption of the untreated cells, as determined by the MTT assay.

**Table S2**

Cytotoxicity in K562 leukemia cell lines induced by exposure for 24 and 48 h to different concentrations of the complexes I-V as assessed by MTT reduction

| Complexes I-V / Concentration | *% Alive cells*[b] | |
|---|---|---|
| | **24h** | **48h** |
| **I** | | |
| 30 μg/ml | 38.1 ± 0.0 | 30.7 ± 0.1 |
| 10 μg/ml | 54.3 ± 0.0 | 37.7 ± 0.2 |
| **II** | | |
| 20 μg/ml | 23.7 ± 0.5 | 8.07 ± 0.1 |
| 5 μg/ml | 34.1 ± 0.2 | 19.1 ± 0.1 |
| **III** | | |
| 100 μg/ml | 25.5 ± 0.3 | 17.4 ± 0.3 |
| 10 μg/ml | 52.6 ± 0.6 | 29.2 ± 0.6 |
| **IV** | | |
| 30 μg/ml | 40.9 ± 0.0 | 27.5 ± 0.2 |
| 10 μg/ml | 93.4 ± 0.1 | 89.9 ± 0.5 |
| **V** | | |
| 100 μg/ml | 82.2 ± 0.3 | 68.3 ± 0.4 |
| 30 μg/ml | 81.7 ± 0.5 | 78.6 ± 0.5 |

[b] % alive cells were determined using the following formula: %alive cells = (mean absorption of sample 100) / mean absorption of the untreated cells, as determined by the MTT assay.

**Table 1**

Percentage (%) of Trypan Blue (-ve) U937 cells when incubated with the complexes **I-V** for 24, 48 and 72 h

| Complexes I-V / Concentration | % (-ve)cells[a] | | |
|---|---|---|---|
| | **24h** | **48h** | **72h** |
| **I** | | | |
| 20 μg/ml | 93.6 ± 0.5 | 89.1 ± 0.9 | 39.7 ± 0.1 |
| 5 μg/ml | 100 ± 0 | 92.5 ± 0.1 | 93.8 ± 0.3 |
| **II** | | | |
| 10 μg/ml | 99.9 ± 0.1 | 89.0 ± 0.6 | 49.2 ± 0.8 |
| 1 μg/ml | 91.9 ± 0.8 | 92.4 ± 0.3 | 89.7 ± 0.5 |
| **III** | | | |
| 100 μg/ml | 97.9 ± 0.3 | 82.4 ± 0.3 | 72.2 ± 0.6 |
| 10 μg/ml | 96.9 ± 0.2 | 81.3 ± 0.3 | 71.4 ± 0.3 |
| **IV** | | | |
| 20 μg/ml | 93.7 ± 0.6 | 78.5 ± 0.6 | 49.2 ± 0.8 |
| 5 μg/ml | 89.8 ± 0.1 | 91.5 ± 0.9 | 89.7 ± 0.5 |
| **V** | | | |
| 50 μg/ml | 94.4 ± 0.1 | 82.4 ± 0.3 | 87.3 ± 0.2 |
| 5 μg/ml | 94.1 ± 0.3 | 83.3 ± 0.1 | 88.3 ± 0.5 |

[a] % -ve cells were determined using the following formula:

%-ve cells=(number of unstained cells/number of total cells)x100. Data are percentage of alive cells ± SD of the each experiment performed in duplicate.

**Table 2.**

Percentage (%) of Trypan Blue (-ve) K562 cells when incubated with the complexes **I-V** for 24 and 48 h

| Complexes I-V / Concentration | *% (-ve)cells[a]* | |
|---|---|---|
| | **24h** | **48h** |
| **I** | | |
| 30 μg/ml | 87.5 ± 0.1 | 69.9 ± 0.1 |
| 10 μg/ml | 99.9 ± 0.1 | 83.3 ± 0.2 |
| **II** | | |
| 20 μg/ml | 92.1 ± 0.3 | 86.5 ± 0.2 |
| 5 μg/ml | 85.7 ± 0.6 | 73.6 ± 0.5 |
| **III** | | |
| 100 μg/ml | 85.4 ± 0.1 | 78.4 ± 0.9 |
| 10 μg/ml | 90.3 ± 0.4 | 86.6 ± 0.3 |
| **IV** | | |
| 30 μg/ml | 91.1 ± 0.3 | 89.2 ± 0.3 |
| 10 μg/ml | 88.9 ± 0.1 | 73.4 ± 0.5 |
| **V** | | |
| 100 μg/ml | 91.5 ± 0.2 | 89.9 ± 0.1 |
| 30 μg/ml | 95.2 ± 0.6 | 91.2 ± 0.1 |

[a] % -ve cells were determined using the following formula:

%-ve cells=(number of unstained cells/number of total cells)x100. Data are percentage of alive cells ± SD of the each experiment performed in duplicate.

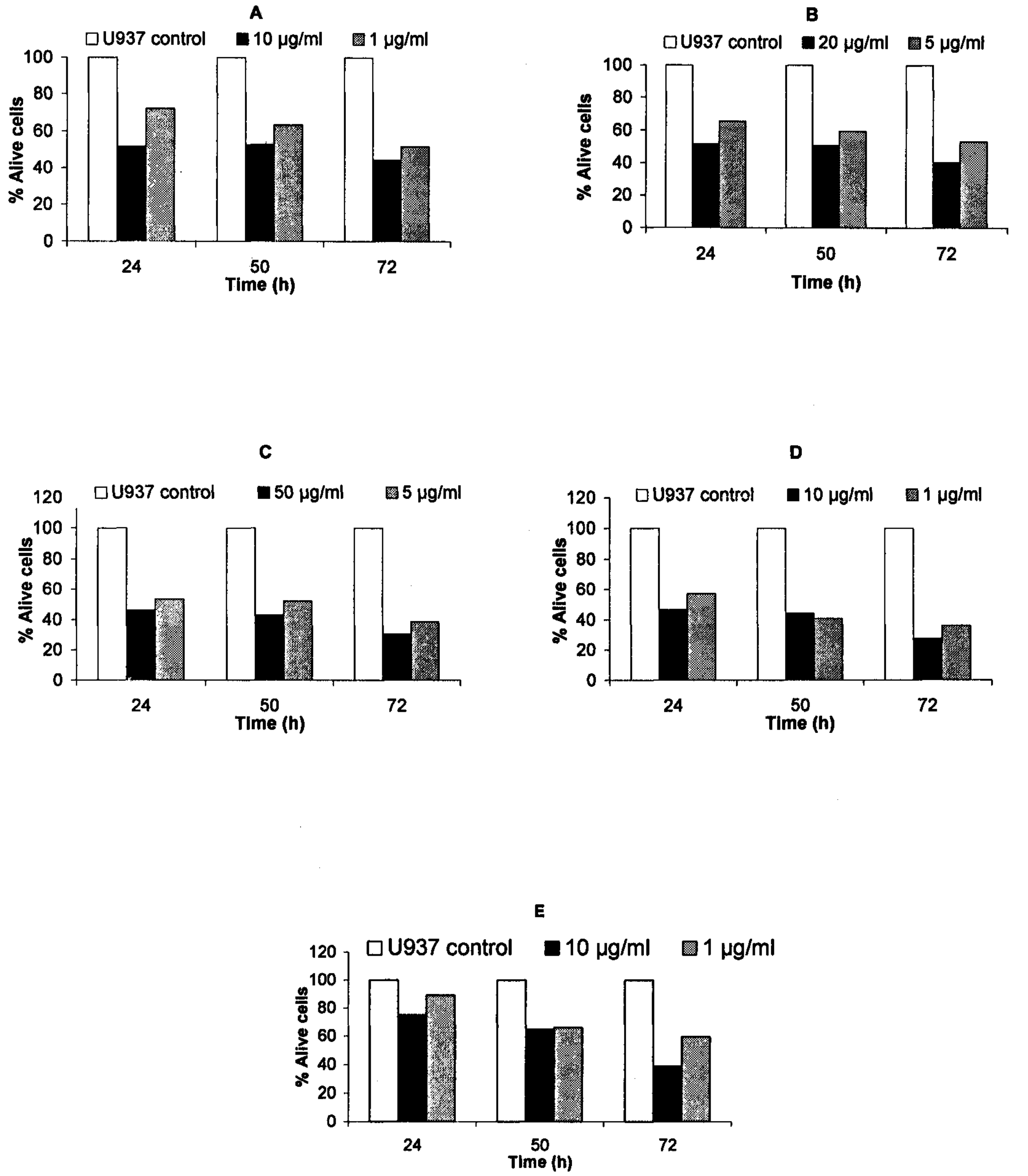

**Fig. 3:** Time- and dose-dependent cytotoxic effects of the complexes A-E on U937 cells. $10\text{-}12 \cdot 10^5$ cells/ml were incubated with the drugs for 24, 50 and 72 hours. Every 24 h aliquot of the cell suspensions were removed and cell viability was evaluated by the MTT colorimetric assay (as described in Section 2.). The data are expressed as a percentage of the control MTT reduction (100%).

It can be seen in Fig. 3 that most of the molybdenum complexes examined have high toxicity in U937 cells which increases with time of incubation. Exception is complex **E**, which does not exhibit high toxicity during the first 24 h of incubation.

The results presented in Table S3 (Supplementary Material) for the MTT assay, show that $IC_{50}$ values for the complexes (**A-E**) in U937 cells were approximately at 10 μM (for **A**) ≈ 20 μM (for **B**) > 50 μM (for **D**) > 50 μM (for **C**) in 24 h and lower than 5 μM and 50 μM (for **E**) after 24 h and 50 h, respectively. From all of the molybdenum complexes tested **E** has the least toxicity, while **C** is the most toxic.

Table 3 presents the percentage of Trypan Blue (-ve) cells in U937 cell lines. The data show that the number of alive (unstained) cells in 24, 50 and 72 h for U937 cells for all the complexes are higher then 85%, which means that none of the complexes, except the complex **C** at higher concentration, exhibits significant necrotic effect on the cell lines tested.

### 4. CONCLUSION

The cytotoxicity tests on selected cobalt and molybdenum complex compounds showed that the cobalt compounds (**I-V**) had low toxicity in U937 cell line, while they exhibited high toxicity in K562 cells. The results obtained by the molybdenum complexes in U937 cells showed that most of the complexes were toxic during the first 24 hours of incubation. These results have confirmed that cobalt complexes are generally active among a series of complexes, then group containing molybdenum. This difference can be due to a lot of factors, as difference in metal centers presented as well as their coordination sphere surroundings.

The cell growth inhibition assays represent the standard criterion for the screening of antitumor compounds. However, this approach does not give direct information on the mechanism of action of the individual drugs, but we can say that these substances behave differently on cells under study probably because they act on different mechanisms. The effects of the complexes to the leukemia cell lines can be due to a number of factors, like induction of apoptosis or differentiation. What is certain in the case of most of the compounds tested is that the toxicity of the compound that enters cells is roughly proportional to the exposure time.

### 5. ACKNOWLEDGEMENTS

This work was supported by the Greek-Serbian collaboration: Ministry of Science, Technology and Development of the Republic Serbia (Project 1318) and General Secretariat for Research and Technology of Greece. We would like to thank Dr Sofija P. Sovilj for providing the complexes and also Mrs Hellinida Thomadaki for her valuable assistance.

**Table S3**

Cytotoxicity in U937 leukemia cell lines induced by exposure for 24, 50 and 72 h to different concentrations of the complexes A-E as assessed by MTT reduction

| Complexes A-E / Concentration | % Alive cells[b] | | |
|---|---|---|---|
| | 24h | 50h | 72h |
| **A** | | | |
| 10 μg/ml | 51.60 ± 0.2 | 52.73 ± 0.3 | 44.31 ± 0.3 |
| 1 μg/ml | 72.09 ± 0.7 | 63.49 ± 0.1 | 51.76 ± 0.0 |
| **B** | | | |
| 20 μg/ml | 51.60 ± 0.1 | 50.70 ± 0.1 | 40.37 ± 0.1 |
| 5 μg/ml | 65.41 ± 0.0 | 59.44 ± 0.1 | 53.21 ± 0.0 |
| **C** | | | |
| 50 μg/ml | 45.93 ± 0.1 | 42.90 ± 0.2 | 30.43 ± 0.1 |
| 5 μg/ml | 53.63 ± 0.6 | 52.26 ± 0.2 | 38.72 ± 0.1 |
| **D** | | | |
| 50 μg/ml | 46.51 ± 0.1 | 44.15 ± 0.3 | 27.54 ± 0.2 |
| 5 μg/ml | 57.12 ± 0.5 | 40.72 ± 0.2 | 36.02 ± 0.1 |
| **E** | | | |
| 50 μg/ml | 74.85 ± 0.1 | 64.90 ± 0.0 | 38.92 ± 0.6 |
| 5 μg/ml | 89.24 ± 0.6 | 66.15 ± 0.1 | 59.57 ± 0.3 |

[b] % alive cells were determined using the following formula: %alive cells = (mean absorption of sample 100) / mean absorption of the untreated cells, as determined by the MTT assay.

**Table 3**

Percentage (%) of Trypan Blue (-ve) U937 cells when incubated with **A-E** complexes for 24, 50 and 72 h

| **Complexes A-E / Concentration** | ***% (-ve)cells***[aa] | | |
|---|---|---|---|
| | **24h** | **50h** | **72h** |
| **A** | | | |
| 10 µg/ml | 91.16 ± 0.8 | 90.82 ± 0.3 | 89.56 ± 0.1 |
| 1 µg/ml | 90.42 ± 0.8 | 91.26 ± 0.9 | 90.24 ± 0.3 |
| **B** | | | |
| 20 µg/ml | 96.51 ± 0.2 | 86.87 ± 0.1 | 83.45 ± 0.2 |
| 5 µg/ml | 90.49 ± 0.3 | 90.18 ± 0.1 | 89.98 ± 0.3 |
| **C** | | | |
| 50 µg/ml | 49.98 ± 0.3 | 16.67 ± 0.2 | 10.25 ± 0.3 |
| 5 µg/ml | 89.61 ± 0.4 | 83.64 ± 0.3 | 80.42 ± 0.6 |
| **D** | | | |
| 10 µg/ml | 87.29 ± 0.3 | 87.08 ± 0.6 | 85.43 ± 0.5 |
| 1 µg/ml | 91.21 ± 0.2 | 91.65 ± 0.1 | 90.32 ± 0.6 |
| **E** | | | |
| 10 µg/ml | 94.23 ± 0.3 | 93.34 ± 0.4 | 92.25 ± 0.1 |
| 1 µg/ml | 94.45 ± 0.6 | 94.82 ± 0.6 | 94.50 ± 0.2 |

[a] % -ve cells were determined using the following formula:

%-ve cells=(number of unstained cells/number of total cells)x100. Data are percentage of alive cells ± SD of the each experiment performed in duplicate.

### 6. REFERENCES

1. D.W. Bombick, P.H. Ayres, K. Putnam, B.R. Bombick and D.J. Doolittle, *Food Chem. Toxicol.*, **36,** 191 (1998)
2. M. Balls, B. Blaauboer, D. Brusick, D. Frazier, D. Lamb, M. Pemberton, C. Reinhardt, M. Roberfroid, H. Rosenkranz, B. Schmid, H. Spielmann, A.L. Stammati and E. Walum, *ATLA*, **18,** 313 (1990)
3. S.P. Sovilj, K. Babić-Samardžija and D. Stojšić, *Spectrosc. Lett.*, **36**, 183 (2003)
4. S.P. Sovilj, G. Vuković, K. Babić-Samardžija, N. Matsumoto, V.M. Jovanović and J. Mrozinski, *Synth. React. Inorg. Met.-Org. Chem.*, **27**, 785 (1999)
5. S.P. Sovilj, D.M. Mitić and V.M. Leovac, *Asian J. Chem.*, **15**, 165 (2003)
6. T. Mosmann, J. *Immunol. M.*, **65**, 55 (1983)
7. H.B. Tada, O. Shino, K. Kurosima, M. Koyama and K.J. Tsukamoto, *J. Immunol. M.*, **93**, 157 (1986)
8. M.B. Hansen, S.E. Nielsen and K. Berg, *J. Immunol. M.*, **119**, 203 (1989)
9. M.B. Hansen, C. Ross and K. Berg, *J. Immunol. M.*, **127,** 241 (1990)

